# One-year outcomes of a digital twin intervention for type 2 diabetes: a retrospective real-world study

**DOI:** 10.1038/s41598-024-76584-7

**Published:** 2024-10-26

**Authors:** Paramesh Shamanna, Ravi Sankar Erukulapati, Ashutosh Shukla, Lisa Shah, Bree Willis, Mohamed Thajudeen, Rajiv Kovil, Rahul Baxi, Mohsin Wali, Suresh Damodharan, Shashank Joshi

**Affiliations:** 1 Bangalore Diabetes Centre, Bangalore, Karnataka 560043 India; 2 Apollo Hospital, Jubilee Hills, Hyderabad, India; 3 Max Hospital & Prana Centre of Integrative Medicine, Gurgaon, Haryana India; 4 Twin Health, Mountain View, CA USA; 5 Twin Health, Mohamed Thajudeen, Research Doctor, Chennai, India; 6 Dr. Kovil’s Diabetes Care Centre, Mumbai, Maharashtra India; 7 Bombay Hospital and Medical Research Centre, Mumbai, India; 8 Sir Ganga Ram Hospital, New Delhi, Delhi India; 9 Hospital and Harvey Speciality Clinic, Coimbatore, India; 10 Department of Diabetology and Endocrinology, Lilavati Hospital and Research center, Mumbai, India

**Keywords:** Artificial intelligence, Diabetes management, Digital twin, Insulin resistance, Type 2 diabetes, Diabetes, Endocrine system and metabolic diseases

## Abstract

This retrospective observational study, building on prior research that demonstrated the efficacy of the Digital Twin (DT) Precision Treatment Program over shorter follow-up periods​​, aimed to examine glycemic control and reduced anti-diabetic medication use after one-year in a DT commercial program. T2D patients enrolled had adequate hepatic and renal function and no recent cardiovascular events. DT intervention powered by artificial intelligence utilizes precision nutrition, activity, sleep, and deep breathing exercises. Outcome measures included HbA1c change, medication reduction, anthropometrics, insulin markers, and continuous glucose monitoring (CGM) metrics. Of 1985 enrollees, 132 (6.6%) were lost to follow-up, leaving 1853 participants who completed one-year. At one-year, participants exhibited significant reductions in HbA1c [mean change: -1.8% (SD 1.7%), *p* < 0.001], with 1650 (89.0%) achieving HbA1c below 7%. At baseline, participants were on mean 1.9 (SD 1.4) anti-diabetic medications, which decreased to 0.5 (SD 0.7) at one-year [change: -1.5 (SD 1.3), *p* < 0.001]. Significant reductions in weight [mean change: -4.8 kg (SD 6.0 kg), *p* < 0.001], insulin resistance [HOMA2-IR: -0.1 (SD 1.2), *p* < 0.001], and improvements in β-cell function [HOMA2-B: +21.6 (SD 47.7), *p* < 0.001] were observed, along with better CGM metrics. These findings suggest that DT intervention could play a vital role in the future of T2D care.

## Introduction

Type 2 diabetes mellitus (T2D) currently affects over 537 million individuals worldwide, with projections indicating that this number could rise to more than 783 million by 2045.^1^ The rapid increase in T2D cases and increasing prevalence among high-risk ethnic groups is largely attributed to aging populations, rising rates of obesity and sedentary lifestyle.^2^ The health implications of T2D are profound, leading to increased risks of cardiovascular diseases, end-stage renal disease (ESRD), retinopathy, and neuropathy.^2^ These projections underscore the urgency for effective and innovative management strategies, such as those demonstrated in recent studies on digital twin (DT) technology for diabetes care​​.^3,4^ Managing T2D effectively requires a multifaceted approach that includes both lifestyle modifications and pharmacological interventions to achieve and maintain long-term metabolic control​​.^2^ Such multifaceted approaches have shown significant improvements in metabolic health parameters, as evidenced by prior studies on digital interventions like the DT Program, which demonstrated improvements in glycemic control and reductions in medication usage​​​​.^3,4^

The UK Prospective Diabetes Study (UKPDS) highlighted the critical role of intensive glucose management in T2D, showing that maintaining a median HbA1c of 7.0% significantly lowered the risk of microvascular complications and myocardial infarction compared to conventional therapy, which resulted in a median HbA1c of 7.9%.^5^ Despite these benefits, data indicate that only about 23.4% of diabetic patients manage to achieve glycemic control within the target range​​.^6^ Current guidelines emphasize the need for rigorous glycemic control to prevent or slow the progression of diabetic complications​​.^7^ The American Diabetes Association (ADA) highlights the critical role of technology in modern diabetes management, including the use of continuous glucose monitoring (CGM) for real-time glucose tracking and advocate for embracing telehealth and digital tools for achieving glycemic control.^7,8^

Low-calorie (LCDs) and very low-calorie diets (VLCDs) are often employed for weight loss and T2D management, but these approaches have significant drawbacks. They often cause nutritional deficiencies, leading to fatigue, hair loss, and weakened immune function due to inadequate intake of essential nutrients like fiber and vitamins.^9^ Muscle mass loss due to insufficient protein is a major concern.^10^ Additionally, VLCDs increase the chance of ventricular arrhythmias, possibly due to nutrient deficiencies that alter cardiac repolarization, increasing the risk of sudden death.^11^ Thus, calorie restriction may not be safe for long-term glycemic control. Similarly, low-carbohydrate diets, which focus on reducing carbohydrate intake to manage blood glucose levels, have shown only modest reductions in the incremental area under the curve (iAUC) for blood glucose by only 16.73%​​.^12^ Despite some success with these diets, long-term adherence and effectiveness remain problematic.^13^

Dietary intake is a central determinant of blood glucose levels, and thus, in order to achieve optimal glucose levels, it is imperative to make food choices that induce normal postprandial glucose response (PPGR). In recent years, various technological interventions have been explored to improve T2D management, including CGM, predictive modelling for PPGR based on the specific foods consumed in each meal, and telehealth.^14–16^ However, these interventions often focus on singular aspects of diabetes care and may not fully integrate the complex factors influencing individual metabolic health​​.

The DT approach represents a significant advancement in T2D management by creating a comprehensive, personalized intervention that addresses the multifaceted nature of the disease. Unlike other technologies that offer generalized solutions, the DT intervention utilizes machine learning algorithms and the Internet of Things (IoT) to create virtual replicas or “twins” of individuals.^17,18^ These digital twins enable predictive modeling and personalized management of T2D by integrating a wide range of data, including CGM, precision nutrition, physical activity, sleep patterns, and stress management through deep breathing exercises​​.^3,4,17,18^

What makes the DT approach unique and potentially more effective than other technological interventions is its ability to provide a highly tailored and dynamic response to an individual’s specific metabolic needs.^3,4,17,18^ The DT system continuously collects and analyzes data from various sensors and inputs, allowing it to offer personalized dietary and lifestyle recommendations that are precisely calibrated to minimize PPGRs and improve overall glycemic control​​. DT platform will suggest the right food to the right participant at the right time. This level of personalization goes beyond the capabilities of traditional CGM and dietary interventions, which often rely on static guidelines that may not account for the individual’s unique metabolic profile.

Technological nudges are effective for behavioral change.^19^ The DT intervention incorporates behavioral nudges and human coaching to enhance engagement and adherence, addressing a common challenge in digital health interventions. By combining the predictive power of AI with real-time feedback and support, the DT approach offers a holistic solution that integrates all aspects of diabetes management, potentially leading to better outcomes in glycemic control, weight management, and medication reduction​​.^4^

The primary research question of this study is: Can a commercially available DT intervention significantly improve glycemic control, reduce the need for anti-diabetic medications, and enhance overall metabolic health in individuals with T2D over the course of one year? We hypothesize that the DT intervention will result in significant improvements in key outcomes, including a reduction in HbA1c levels, a decrease in the use of anti-diabetic medications, and improvements in insulin sensitivity and β-cell function. These outcomes are expected to be superior to those achieved with traditional diet and lifestyle interventions, highlighting the potential of the DT approach in T2D management.

## Methods

### Study design and patient population

This retrospective observational study evaluated the outcomes of a DT intervention for T2D in a real-world setting. The study builds on a previously established framework for evaluating DT interventions in diabetes care, which has demonstrated success in short-term outcomes such as reductions in glycemic variability and medication use​​.^3^ The study included 1853 participants who enrolled in the DT program and completed at least one year of follow-up. Participants were recruited from January 2022 to December 2022, with the follow-up period concluding in December 2023. Participants were recruited from multiple diabetes care centers across India, including multidisciplinary diabetic clinics and other healthcare facilities. Recruitment strategies included direct outreach to eligible patients, referrals from healthcare providers, and informational sessions about the study. Participants were eligible for inclusion if they were diagnosed with T2D, aged between 18 and 80 years, and had the capability to adhere to the program for at least one year. Exclusion criteria included elevated liver enzymes (aspartate transaminase or alanine transaminase levels three times above the upper limit of normal), reduced renal function (estimated glomerular filtration rate < 45 ml/min/1.73 m^2^), and recent cardiovascular events (myocardial infarction, stroke, or angina within the previous three months). There were no restrictions on HbA1c levels, T2D duration, or baseline T2D medications, allowing us to evaluate the DT intervention across diverse glycemic controls and disease stages. Patients with T2D complications were included unless they met exclusion criteria, ensuring a real-world representation of T2D patients with comorbidities. The study protocol was approved by the Medisys Clinisearch Ethical Review Board, adhering to the principles of the Declaration of Helsinki and its amendments. All participants provided informed consent for the use of their data in the study.

### DT intervention

Participants in the DT program were provided with several digital health devices, including a continuous glucose monitor (CGM; Abbott FreeStyle Libre Pro®), sensor watch (Fitbit Charge 2®) to track activity and resting heart rate and smart scale (Powermax® BCA-130 Bluetooth Smart Scale). A clinical history and laboratory profile were obtained at enrollment, then dietary and other data were gathered using the DT sensors and smartphone App (WBDT; Whole Body Digital Twin®), with all information securely transmitted through a cellular network.

Demographic, clinical, and medication history were obtained at entry into the program, including age, gender, diabetes duration, weight, and BMI. At 1-day post-enrollment and 3-month intervals, fasting phlebotomy was performed for biochemical markers. Participants used the DT mobile app to log their daily dietary intake, including details such as the type, quantity, and time of food consumption. The app enabled participants to search for foods in a comprehensive database, which includes specific details of macronutrients, micronutrients, and effects on 4gut microbiome, prepared using USDA’s FoodData Central.^3,^ The DT app is designed for simplicity and ease, with a non-intrusive nudge system tailored to enhance user experience and engagement.

Central to this intervention was deploying a CGM, which the participants use continuously. The real-time data derived from the CGM and the extensive food logs compiled by the participants constitute the foundational data set for the AI model. This AI model used advanced machine learning methodologies, including gradient-boosted trees and neural networks. It processed a multifaceted array of over 200 features, encompassing sensor data, metabolic health parameters, and nutritional information, to predict PPGRs.

Using this extensive dataset, the DT can predict individual-specific PPGRs. Foods were classified within the system using a color code: red indicated foods to avoid, yellow suggested moderation, and green signified recommended foods. This system assisted in guiding dietary choices by offering lower PPGR alternatives for foods that initially present high PPGR, facilitated more tailored and effective nutritional management.^4^

The study employed AI-driven nudges to encourage healthier food choices and specific exercise and lifestyle habits. Participants initially aimed for 5,000 steps/day, increased to 10,000, 20-minute resistance exercises three times a week, reduced sedentary time of < 8 h, 7–8 h of sleep, and daily deep breathing exercises for stress reduction. The app conveyed 15–20 context-appropriate nudges daily. Certified human health coaches supplemented these nudges thrice weekly, tapering to weekly, with on-demand support after that, ensuring consistent member engagement throughout the program.

The program had three phases: (1) a 90-day “restricted” phase limited foods with high PPGRs; (2) a 90-day “re-introduction” phase gradually allowed these foods to be reintroduced; and (3) a “maintenance” phase with AI-guided dietary advice for the rest of the program. Participants ate until satiated in each phase.

Anti-diabetic medications were down-titrated and discontinued step-wise based on standard protocol as follows: Participants who were already on insulin at baseline had their bolus insulin stopped and basal doses reduced by 50%, then reduced further and discontinued based on CGM readings. Participants on the medications, including metformin, sulfonylureas, thiazolidinediones, alpha-glucosidase inhibitors, glucagon-like peptide-1 receptor agonists (GLP1ras), and/or sodium-glucose cotransporter-2 inhibitors (SGLT2is) were down-titrated and discontinued based on CGM readings. The adjustment of these medications was supervised by physicians.

### Outcome measures

HbA1c and the following parameters were assessed at baseline and one year after enrollment: weight (kg), body mass index (BMI, kg/m^2^), fasting plasma glucose (FPG, mg/dl), homeostatic model assessment of insulin resistance (HOMA2-IR),^20^ homeostatic model assessment of beta cell function (HOMA2-B).^20^ CGM Metrics assessed were Time in Range (TIR), Time Above Range-1 and 2 (TAR-1, TAR-2), Time Below Range-1 and 2 (TBR-1, TBR-2) were assessed at baseline and one year after enrollment. TIR refers to the percentage of glucose levels within the target range (between 70 mg/dl and 180 mg/dl). For determination of TAR, hyperglycemia was subdivided into level 1 (glucose 181–250 mg/dl) and level 2 (glucose > 250 mg/dl); for determination of TBR, hypoglycemia was subdivided into level 1 (glucose 54–69 mg/dl) and level 2 (glucose < 54 mg/dl).^21,22^

### Statistical analysis

In this retrospective analysis, the study evaluated outcomes from a one year commercial program using an observational design. Continuous variables are scrutinized for normality through graphical methods and the Shapiro-Wilk’s test, subsequently being reported as means with standard deviations (SD). Categorical variables are presented as counts and percentages. For the analysis of one year program outcomes, quantitative data are evaluated using appropriate statistical methods. Continuous variables are analyzed using paired t-test. This approach facilitated the comparison of pre-and post-program measurements within the same cohort. The study’s statistical analyses were conducted using SPSS version 28.0, and a p-value of less than 0.05 was considered statistically significant. To address potential confounding factors and reduce selection bias, a multivariable linear regression analysis was conducted. Multivariable modeling was conducted only for HbA1c, as it was designated as the primary outcome of the study. This analysis adjusted for baseline covariates including age, gender, diabetes duration, baseline HbA1c, BMI, HOMA2-IR, and HOMA2-B to isolate the impact of the DT intervention on HbA1c changes at one year, ensuring a more accurate efficacy assessment. In the multivariate regression, all predefined covariates were retained in the model regardless of statistical significance to assess their independent effects on HbA1c changes at one year. No step-wise variable selection was used to ensure consistency across analyses, as the primary goal was adjusting for potential confounders rather than model parsimony. Collinearity analysis was also performed for all variables.

## Results

### Baseline demographics and clinical characteristics

A total of 1985 participants were enrolled in the DT Program between January and December 2022. After excluding 132 participants (6.6%) due to loss to follow-up, 1853 participants were included in the final analysis. The mean age was 50.9 years (SD 9.9 years), and the mean duration of diabetes was 6.7 years (SD 6.2 years) at enrollment. The cohort was balanced in terms of age distribution, with 46.5% of participants aged < 50 years and 53.5% aged ≥ 50 years, providing an opportunity to evaluate the intervention’s efficacy across a broad age spectrum. Similarly, 72.5% of participants had a diabetes duration of < 10 years, while 27.5% had a duration of ≥ 10 years, reflecting a mix of early and long-standing T2D cases. The population included a high prevalence of obesity (63.1%), hypertension (38.2%), and non-alcoholic fatty liver disease (NAFLD) (83.4%). A smaller proportion had atherosclerotic cardiovascular disease (6.1%) or chronic kidney disease (1.9%), mirroring the complex comorbidities typically encountered in real-world diabetes management (Table 1). These baseline characteristics establish a comprehensive and diverse participant profile, enabling the evaluation of the DT intervention’s effectiveness across various sub-groups.

**Table 1:** Baseline characteristics of DT program participants*.

Parameters	Subcategory	Values
Number of Participants		1853
Age, years		50.9 (9.9)
	< 50 years, no. (%)	861(46,5%)
	>= 50 years, no. (%)	992 (53.5%)
Female, no. (%)		379, (20.5%)
Duration of Diabetes, years		6.7(6.2)
	< 10 years, no. (%)	1343 (72.5%)
	>=10 years, no. (%)	510 (27.5%)
BMI, kg/m^2^		27.0 (4.6)
	< 25 kg/m^2^, no. (%)	1169 (63.1%)
	>=25 kg/m^2^, no. (%)	684 (36.9%)
HbA1c, %		8.1 (1.7)
Fasting Plasma Glucose, mg/dl		151.8 (61.8)
Number of T2D medications		1.9 (1.4)
Participants on insulin, no. (%)		166 (8.9%)
Participants on metformin, no. (%)		1328 (71,7%)
Participants on DPP4 inhibitors, no. (%)		744 (40.2%)
Participants on Sulfonylurea, no. (%)		721 (39.6%)
Participants on SGLT2 inhibitors, no. (%)		274 (14.8%)
Participants on GLP-1 receptor agonist, no. (%)		8 (0.4%)
Participants not on T2D medications, no. (%)		337 (18.2%)
HOMA2-IR		2 (1.2)
HOMA2-B		68.3 (44.3)
Participants with Obesity, no. (%)		684 (36.9%)
Participants with Hypertension, no. (%)		707 (38.2%)
Participants with Dyslipidemia, no. (%)		682 (36.8%)
Participants with ASCVD, no. (%)		113 (6.1%)
Participants with NAFLD, no. (%)		1545 (83.1%)
Participants with CKD, no. (%)		36 (1.9%)

*Data are mean (SD), no. (%).

Abbreviations: ASCVD – Atherosclerotic cardiovascular disease; BMI – body mass index (kg/m^2^); CKD – chronic kidney disease; DT – digital twin; DPP4 – Dipeptidyl peptidase-4; GLP-1 – Glucagon-like peptide-1; HbA1c – hemoglobin A1c; HOMA2-B – homeostatic model assessment 2 for β-cell function; HOMA2-IR – homeostatic model assessment 2 of insulin resistance; NAFLD – Non-alcoholic fatty liver disease; SGLT2 – Sodium-glucose cotransporter-2; T2D – type 2 diabetes.

### Glycemic control

The one-year DT intervention was associated with a substantial improvement in glycemic control, with mean HbA1c decreasing from 8.1% (SD 1.7%) at baseline to 6.3% (SD 0.5%) at one year (*p* < 0.001). The magnitude of this reduction [mean change: -1.8% (SD 1.7%); *p* < 0.001] is clinically significant, indicating the intervention’s effectiveness in achieving meaningful glycemic improvements even among participants with a high baseline HbA1c.

**Fig. 1:**
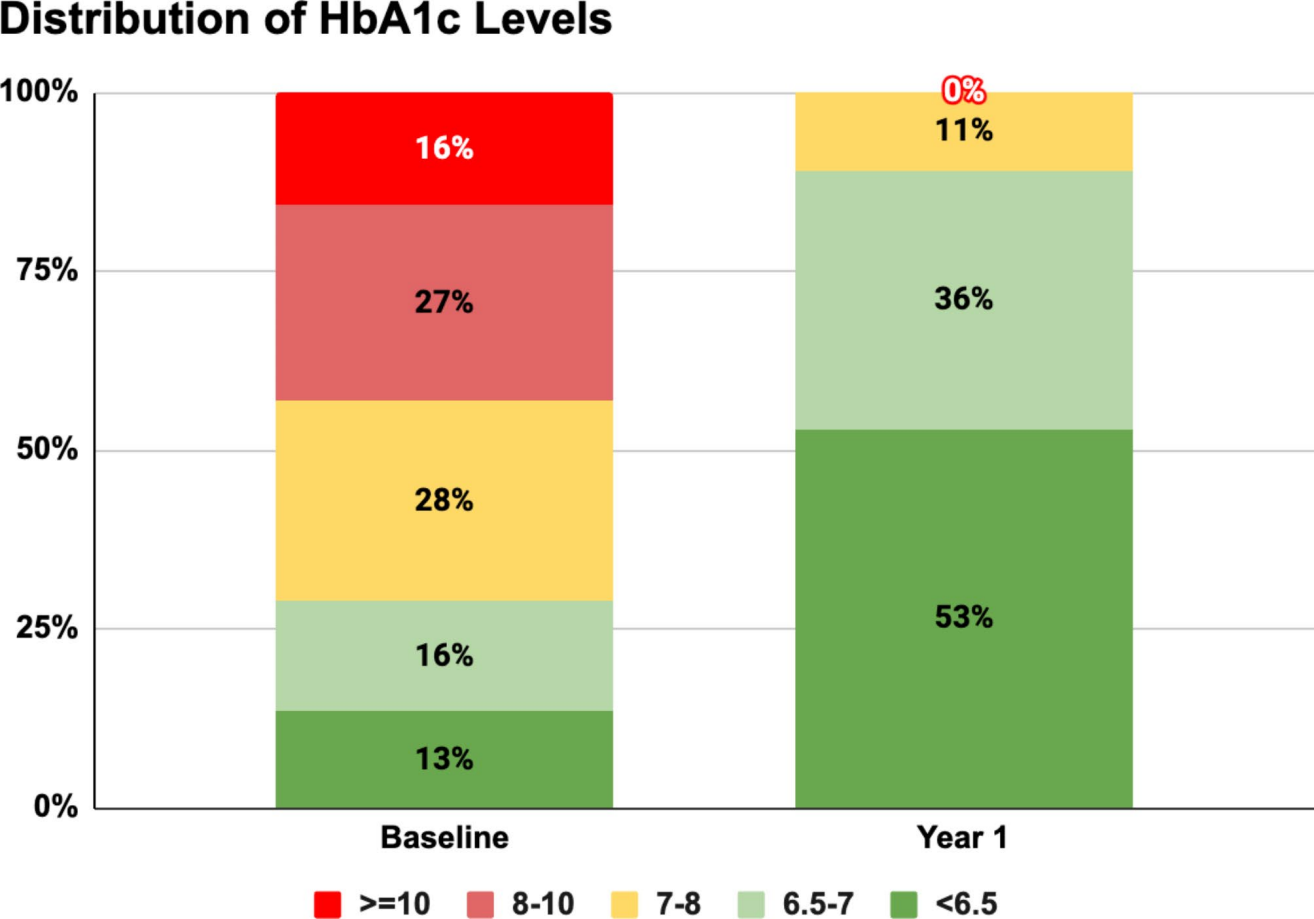
Distribution of HbA1c levels after one year among DT Intervention participants*. Abbreviations: DT – digital twin; HbA1c – hemoglobin A1c.

At the end of the one-year, 89.0% (1650 of 1853) of participants achieved HbA1c levels below 7%, a key target in diabetes management. 81.5% (1511 participants) achieved this target with either no medications or metformin alone, demonstrating the intervention’s potential to reduce medication dependence. Of the total participants, 60.3% (1117) attained HbA1c < 7% without using any T2D medicines, while an additional 21.3% (394) reached this target using metformin as monotherapy (Fig. 1). These outcomes underscore the program’s effectiveness in enabling drug-free or minimal-drug management for glycemic control.

### CGM metrics

**Fig. 2:**
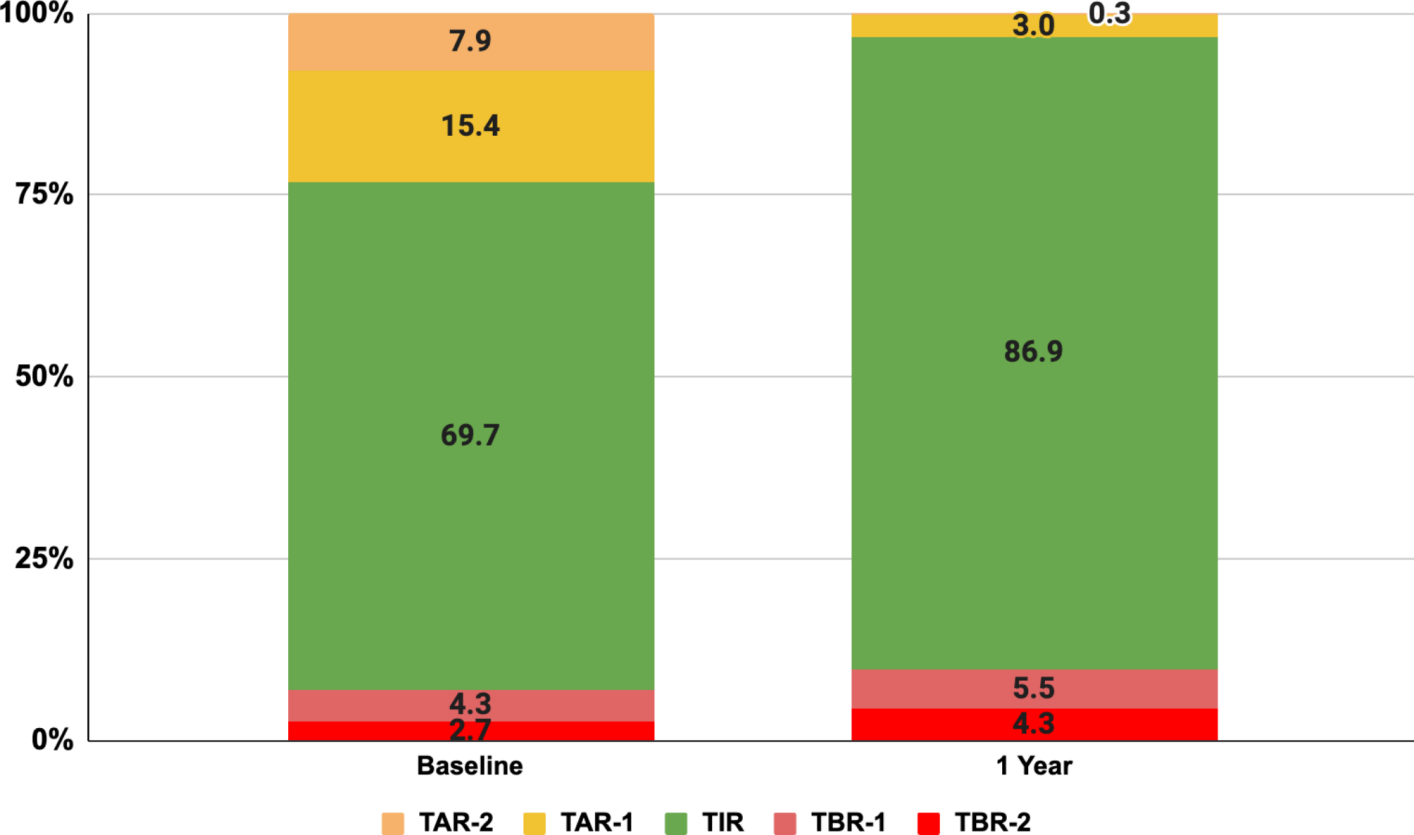
CGM metrics after one year among DT Intervention participants*. Abbreviations: CGM – continuous glucose monitor; DT – digital twin; TAR-1 - time above range level 1 (interstitial glucose 181–250 mg/dl); TAR-2 - time above range level 2 (interstitial glucose > 250 mg/dl); TBR-1 - time below range level 1 (interstitial glucose 54–69 mg/dl); TBR-2 - time below range level 2 (interstitial glucose < 54 mg/dl); TIR - time in range (interstitial glucose 70–180 mg/dl).

The significant reductions in HbA1c were substantiated by improvements in time-based CGM metrics, which provide a detailed assessment of glycemic stability. TIR increased significantly from 69.7% (SD 30.7%) at baseline to 86.9% (SD 24.5%) at one year (*p* < 0.001), indicating better overall glycemic control. TAR-1 and TAR-2 both showed significant reductions, reflecting fewer hyperglycemic episodes. Specifically, TAR-2 decreased from 7.9% (SD 19.9%) to 0.3% (SD 3.4%) (*p* < 0.001), indicating a reduction in severe hyperglycemia (Fig. 2). Changes in TBR metrics were evaluated to assess the risk of hypoglycemia. TBR-1 increased slightly from 4.3% (SD 9.3%) to 5.5% (SD 14.2%) (*p* = 0.001), which was clinically insignificant. However, there was a significant increase in TBR-2 from 2.7% (SD 9.9%) to 4.3% (SD 16.0%) [mean change: +1.6% (SD 18.7%); *p* < 0.001]. Despite these increases, no symptomatic or severe hypoglycemic episodes were reported during the intervention, suggesting that the DT Program achieves stringent glycemic control without substantial risk of clinically dangerous hypoglycemia.

### Reduction in antidiabetic medications

A significant reduction in antidiabetic medication use was observed over the one-year DT intervention period. The mean number of medications per participant decreased from 1.9 (SD 1.4) at baseline to 0.5 (SD 0.7) at one year (*p* < 0.001), reflecting a 74% reduction in the pharmacotherapy burden. This outcome suggests that improved glycemic control was primarily driven by non-pharmacological measures, including dietary modifications and lifestyle interventions, facilitated by the DT Program.

**Fig. 3:**
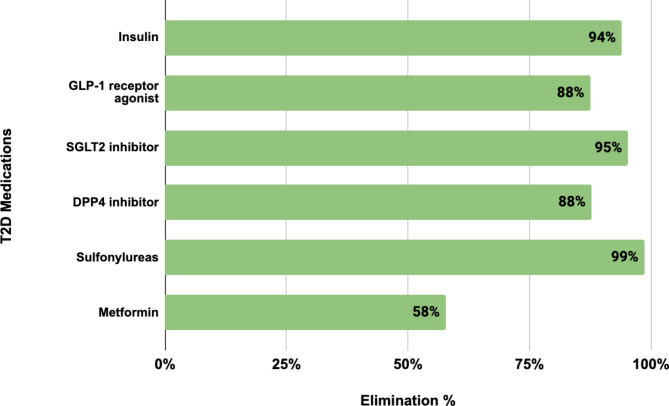
T2D Medication elimination after one year among DT Intervention participants*. Abbreviations: DT – digital twin; DPP4 – Dipeptidyl peptidase-4; GLP-1 – Glucagon-like peptide-1; SGLT2 – Sodium-glucose cotransporter-2; T2D – type 2 diabetes.

**Fig. 4:**
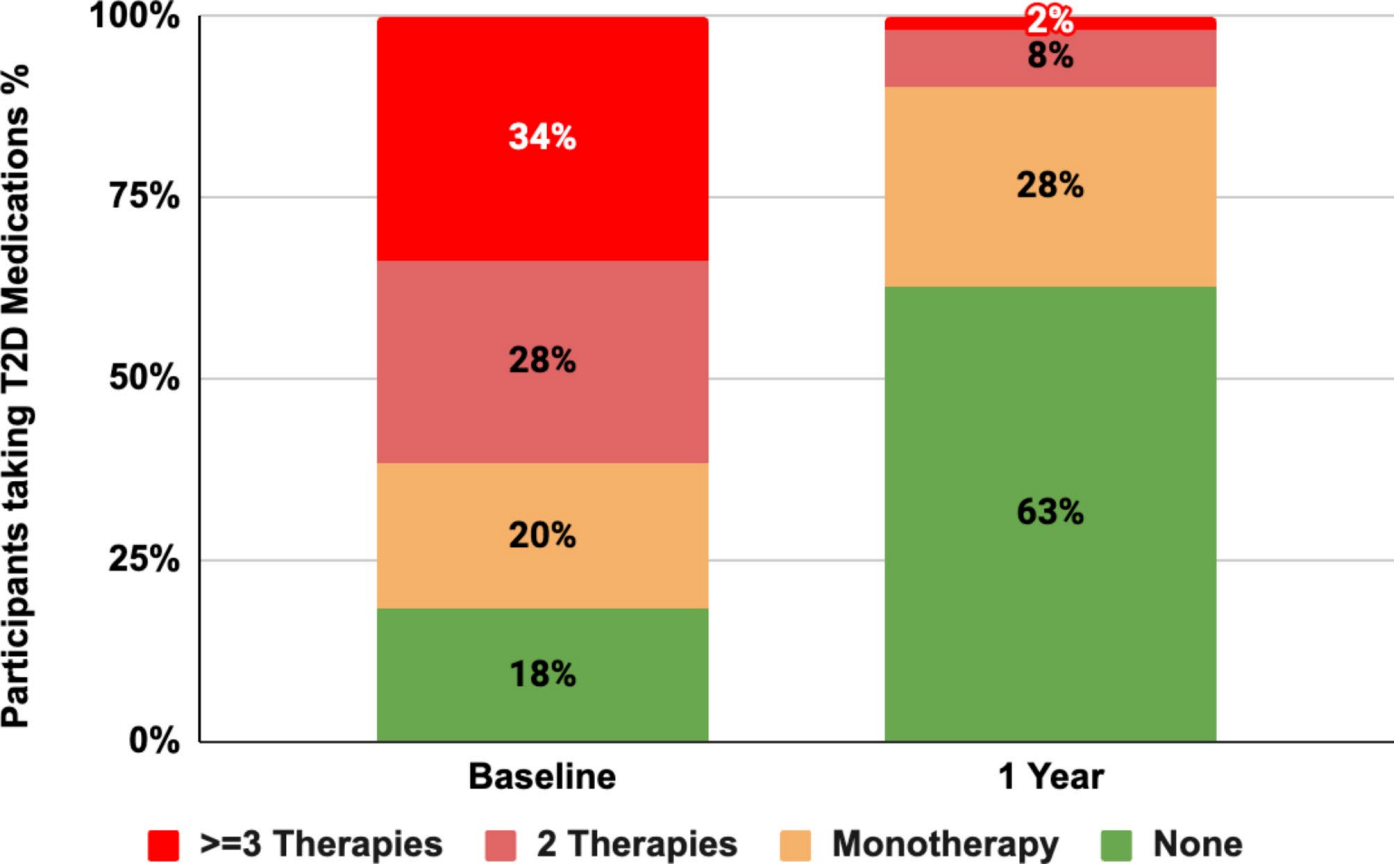
Participants taking T2D Medications after one year of DT Intervention*. Abbreviations: DT – digital twin; T2D – type 2 diabetes.

The most significant changes were seen in the use of insulin and sulfonylureas. At baseline, 166 participants (8.9%) were on insulin therapy, with a mean daily dose of 31.7 units (SD 23.7 units). After one year, 94% discontinued insulin, leaving only 10 participants (0.5%) still on therapy, with a significantly reduced mean dose of 1.9 units (SD 8.4 units). Among the 10 participants who continued insulin, the mean dose decreased from 52.6 units (SD 38.3 units) to 31.0 units (SD 16.7 units). Those initially on insulin had a mean HbA1c of 8.8% (SD 1.7%) at baseline, which dropped to 6.5% (SD 0.5%) at one year, compared to an overall reduction in the study cohort from 8.1% (SD 1.7%) to 6.3% (SD 0.5%). These findings demonstrate the DT intervention’s effectiveness in reducing insulin dependence while achieving significant glycemic improvement (Fig. 3). Sulfonylurea use dropped by 99% (from 721 participants at baseline to 10 participants at one year), and DPP4 inhibitor usage decreased by 88% (Fig. 3). By the end of one year, 62.7% of participants were able to maintain glycemic control without any pharmacotherapy (Fig. 4), demonstrating the DT Program’s potential to significantly reduce or eliminate medication use in a large proportion of participants.

### Metabolic and anthropometric changes

In addition to improvements in glycemic parameters, significant reductions in body weight and other metabolic markers were observed. Mean body weight decreased from 76.9 kg (SD 14.1 kg) at baseline to 72.1 kg (SD 13.4 kg) at one year (*p* < 0.001), representing a mean weight loss of 4.8 kg (SD 5.7 kg). This was accompanied by a significant decrease in BMI from 27.0 kg/m^2^ (SD 4.6 kg/m^2^) to 25.3 kg/m^2^ (SD 4.3 kg/m^2^) (*p* < 0.001), with 70.4% of participants who had a BMI ≥ 25 kg/m^2^ at baseline achieving normalization.

FPG significantly decreased from 151.8 mg/dL (SD 61.8 mg/dL) to 119.4 mg/dL (SD 25.0 mg/dL) [mean change: -32.4 mg/dL (SD 60.8 mg/dL); *p* < 0.001]. Insulin resistance, measured by HOMA2-IR, showed a slight reduction from 2.0 (SD 1.2) to 1.9 (SD 0.9) (*p* < 0.001). In parallel, β-cell function, measured by HOMA2-B, increased significantly from 68.3 (SD 44.3) to 89.9 (SD 41.9) (*p* < 0.001), suggesting improved pancreatic function (Table 2). These findings demonstrate that the intervention not only led to better glycemic control but also resulted in broader metabolic benefits.

**Table 2:** Changes in metabolic and anthropometric parameters after one year among T2D participants in the DT Intervention*^†^.

Outcome Measures	Baseline	One Year	Change	*P*-Value^$^
Weight, kg	76.9(14.1)	72.1(13.4)	-4.8(5.7)	**< 0.001**
BMI, kg/m2	27(4.6)	25.3(4.3)	-1.7(2)	**< 0.001**
HbA1c, %	8.1(1.7)	6.3(0.5)	-1.8(1.7)	**< 0.001**
Number of T2D medications	1.9(1.4)	0.5(0.7)	-1.5(1.3)	**< 0.001**
FPG, mg/dl	151.8(61.8)	119.4(25)	-32.4(60.8)	**< 0.001**
HOMA2-IR	2(1.2)	1.9(0.9)	-0.1(1.2)	**< 0.001**
HOMA2-B	68.3(44.3)	89.9(41.9)	21.6(47.7)	**< 0.001**
TIR, %	69.7(30.7)	86.9(24.5)	17.2(38.2)	**< 0.001**
TAR-1, %	15.4(19.6)	3(8.3)	-12.5(20.6)	**< 0.001**
TAR-2, %	7.9(19.9)	0.3(3.4)	-7.6(20)	**< 0.001**
TBR-1, %	4.3(9.3)	5.5(14.2)	1.3(16.6)	**0.001**
TBR-2, %	2.7(9.9)	4.3(16)	1.6(18.7)	**< 0.001**

* Data are mean (SD). ^$^paired t-test.

† Figures in **bold** are significant with p-value < 0.05. Abbreviations: BMI – body mass index (kg/m^2^); FPG - fasting plasma glucose; HbA1c – hemoglobin A1c; HOMA2-B - homeostatic model assessment 2 of beta cell function; HOMA2-IR – homeostatic model assessment 2 of insulin resistance; TAR-1 - time above range level 1 (interstitial glucose 181–250 mg/dl); TAR-2 - time above range level 2 (interstitial glucose > 250 mg/dl); TBR-1 - time below range level 1 (interstitial glucose 54–69 mg/dl); TBR-2 - time below range level 2 (interstitial glucose < 54 mg/dl); TIR - time in range (interstitial glucose 70–180 mg/dl); kg – kilogram; T2D – type 2 diabetes; DT – digital twin.

### Multivariable regression analysis

To identify factors associated with HbA1c changes at one year, a multivariable regression analysis was performed, incorporating baseline characteristics such as age, gender, diabetes duration, baseline HbA1c, BMI, HOMA2-IR, and HOMA2-B (Table 3). The model explained 10.2% of the variance in HbA1c levels (R^2^ = 0.102, adjusted R^2^ = 0.098). Baseline HbA1c was the strongest predictor of HbA1c change, with a one-unit increase at baseline resulting in a 0.0553 unit greater reduction at one year (*p* < 0.001). Similarly, longer diabetes duration was associated with smaller reductions in HbA1c (β = 0.0147 per year; *p* < 0.001). Higher baseline β-cell function (HOMA2-B) was linked to greater HbA1c improvement (β = -0.0010 per unit; *p* = 0.004). These findings highlight the influence of initial glycemic status and disease duration on the intervention’s efficacy, providing insights into patient subgroups that may benefit most from the DT Program.

**Table 3:** Multivariable regression analysis of baseline factors affecting HbA1c changes at one year among T2D participants in the DT intervention.

Variable	Coefficient (β)	*P*-Value	95% Confidence Interval
**Age**	0.0035	0.007	0.001 to 0.006
**Gender (Male)**	-0.0575	0.050	-0.115 to 0.00009
**Duration of Diabetes**	0.0147	< 0.001	0.011 to 0.019
**Baseline HbA1c**	0.0553	< 0.001	0.039 to 0.071
**Baseline BMI**	0.0030	0.271	-0.002 to 0.008
**Baseline HOMA2-IR**	0.0038	0.730	-0.018 to 0.026
**Baseline HOMA2-B**	-0.0010	0.004	-0.002 to -0.0003

Abbreviations: BMI – body mass index (kg/m^2^); HbA1c – hemoglobin A1c; HOMA2-B - homeostatic model assessment 2 of beta cell function; HOMA2-IR – homeostatic model assessment 2 of insulin resistance; T2D – type 2 diabetes; DT – digital twin.

Discussion.

The results of this real-world, retrospective analysis of 1853 patients enrolled in a DT commercial program for T2D over one year demonstrate significant clinical improvements in multiple parameters. A noteworthy finding is that 89.0% of participants achieved an HbA1c level of less than 7%. This reduction in HbA1c, along with substantial decreases in medication usage and improvements in metabolic health markers, aligns well with our study’s hypothesis and objectives. These results are particularly significant as achieving HbA1c levels below 7% is a widely recognized goal in T2D management, indicative of a lower risk of T2D-related complications.^5^ A decrease of 1% in HbA1c levels was linked with a 37% lower risk of microvascular complications and a 21% reduction in the risk of any diabetes-related endpoint or death.^5^

In evaluating the effectiveness of the DT intervention for managing T2D, the current study demonstrated an HbA1c reduction of 1.8%, closely aligning with the 1.9% decrease observed after 90 days in a previous study.^3^ Additionally, the DT intervention in a randomized controlled trial (RCT) reported a more substantial 2.9% reduction in HbA1c levels.^4^ These results suggest that while real-world applications of the DT intervention consistently yield significant reductions in HbA1c, the more controlled conditions of an RCT can facilitate even greater improvements. When comparing the DT intervention to other diabetes management strategies, it becomes clear that the DT approach offers superior outcomes. For example, the Virta Health program, employing a ketogenic diet approach, reported a 1.3% decrease in HbA1c levels.^23^ However, the outcomes that Virta achieved were in large part due to the restrictive ketogenic diet versus the liberal and personalized diet used in our program. The Look AHEAD trial, focusing on intensive lifestyle interventions, observed a 0.7% reduction in HbA1c levels and 72.7% of participants achieving an HbA1c below 7%.^24^ The Diabetes Remission Clinical Trial (DiRECT) trial, utilizing Total Diet Replacement (TDR), achieved a 0.9% reduction in HbA1c levels.^25^ The digital intervention based on the Low-Carb Program reported HbA1c reductions of 1.2%,^26^ the Weight Watchers program reported HbA1c reductions of 0.75%,^27^ and the Why WAIT program, utilizing liquid meal replacements, reported that 21.6% of their participants had an HbA1c less than 6.5%.^28^

This comparative analysis offers insights into HbA1c reductions across various T2D management interventions, underscoring the varied effectiveness of these strategies. The DT intervention’s success can be attributed to its unique integration of advanced technology and personalized health coaching. The CGM data, combined with machine learning algorithms, provided real-time, individualized dietary and lifestyle recommendations. This level of personalization, coupled with regular behavioral nudges and human coaching, facilitated sustained engagement and adherence to healthier habits.^3,4^ This holistic and dynamic approach to T2D management addresses the multifaceted nature of T2D and offers a more comprehensive solution compared to traditional interventions.

Multivariable regression analysis was performed to overcome selection bias by adjusting for key baseline covariates and to identify factors influencing HbA1c changes in participants of the DT intervention. The analysis reveals key factors affecting HbA1c changes. Older age is linked to slightly higher HbA1c levels at one year, indicating that older adults may need more tailored support. Gender differences are minimal, with males showing marginally lower HbA1c levels, suggesting a potential need for gender-specific adjustments. Longer diabetes duration correlates with higher HbA1c levels, highlighting the challenge of managing long-term diabetes and the need for early intervention. Higher baseline HbA1c strongly predicts higher levels at one year, emphasizing the challenge of managing patients with high initial HbA1c. Baseline BMI and HOMA2-IR are not significantly related to HbA1c changes, suggesting the DT intervention’s broad applicability across different BMI and insulin resistance profiles. Better baseline β-cell function (HOMA2-B) is associated with lower HbA1c, highlighting the importance of β-cell health. Overall, the DT intervention effectively reduces HbA1c levels underscoring the importance of personalization and early intervention. This analysis helps minimize selection bias by adjusting for baseline differences, attributing HbA1c changes more accurately to the DT intervention.

The data on medication elimination and reduction, particularly for GLP-1 receptor agonists, SGLT2 inhibitors, and DPP4 inhibitors, highlights the potential of the DT intervention to alleviate some of the pharmacological burden for individuals with T2D (Fig. 3). This is of importance, especially in the context of pharmacoeconomics, as it reduces the reliance on high-cost T2D medications.

Multiple studies have used TIR as an indicator of glucose control to compare different interventions for T2D management.^29^ A study using continuous calorie-restricted diet and time-restricted intermittent fasting demonstrated an improvement in TIR by 28–30%.^30^ Another study comparing a glycemia-targeted specialized supplement to a standardized breakfast found no significant differences in TIR.^31^ A study on low-carbohydrate versus low-fat breakfasts in T2D patients reported significant improvements in TIR with the low-carbohydrate breakfast.^32^ However, a study investigating low-carbohydrate diet scores found no significant association with TIR or other glycemic control indices.^33^ In our study, DT participants spent significantly more time in target glucose levels (86.9%) and less time at very high glucose levels. Similar to previous studies,^34^ CGM-derived TBR metrics tended to overestimate the severity of participant-reported hypoglycemia. Although the DT group showed more low interstitial glucose readings by CGM, these were not linked to severe or symptomatic hypoglycemia. Notably, patients with high TBR values were not on insulin or sulfonylureas at the time. Participants were closely monitored, and any hypoglycemic readings prompted immediate review and adjustments by the healthcare team. The DT intervention includes real-time alerts and nudges to help participants manage potential hypoglycemia while maintaining overall glycemic control (Table 2, Fig. 2). These findings highlight the effectiveness and safety of the DT intervention, demonstrating superior outcomes compared to other dietary interventions by integrating precise nutrition, activity, and sleep management through advanced algorithms and real-time feedback.

The strength of this real-world study lies in its comprehensive approach, demonstrating the effectiveness and utility of the DT intervention across diverse populations with a large sample size. Our findings show significant improvements in glycemic control, reduction in anti-diabetic medication use, and overall metabolic health among participants, highlighting the potential of the DT intervention in managing T2D. However, we acknowledge several limitations. The non-randomized participant selection introduces potential selection bias. The one-year observational period limits long-term sustainability assessment. The study design may not account for all confounding variables, and the absence of a control group prevents definitive attribution of improvements solely to the DT intervention. This study evaluates the real-world effectiveness of the DT for T2D, reflecting variations in patient adherence not seen in our previously published RCT,^4^ which provided stronger evidence of its efficacy in a controlled setting.

## Conclusion

The study demonstrates that the Digital Twin intervention significantly improves glycemic control, reduces the need for anti-diabetic medications, and enhances various aspects of metabolic health in individuals with T2D over a one-year period. These results suggest that the DT approach could play a vital role in the future of T2D management by offering a comprehensive, personalized, and technologically advanced solution. With continued investigation and refinement, the DT intervention holds promise as a transformative tool for managing and improving the quality of life for individuals living with T2D.

## Data Availability

The datasets analysed during the current study are available from the corresponding author on reasonable request.

## References

[1] International Diabetes Federation. IDF Diabetes Atlas, 10th edn. Brussels, Belgium: 2021. Available at: https://www.diabetesatlas.org

[2] Marín-Peñalver, J. J., Martín-Timón, I., Sevillano-Collantes, C. & Del Cañizo-Gómez, F. J. Update on the treatment of type 2 diabetes mellitus. *World J Diabetes.* **7**(17), 354–395 (2016). 27660695 10.4239/wjd.v7.i17.354 PMC5027002

[3] Shamanna, P. et al. Retrospective study of glycemic variability, BMI, and blood pressure in diabetes patients in the Digital Twin Precision Treatment Program. *Scientific Reports.* **11**(1), 14892 (2021). 34290310 10.1038/s41598-021-94339-6 PMC8295289

[4] Joshi, S. et al. Digital twin-enabled personalized nutrition improves metabolic dysfunction-associated fatty liver disease in type 2 diabetes: Results of a 1-year randomized controlled study. *Endocr. Pract.* **29**(12), 960–970 (2023). 37778441 10.1016/j.eprac.2023.08.016

[5] Stratton, I. M. et al. Association of glycaemia with macrovascular and microvascular complications of type 2 diabetes (UKPDS 35): Prospective observational study. *BMJ.* **321**(7258), 405–412 (2000). 10938048 10.1136/bmj.321.7258.405 PMC27454

[6] Borgharkar, S. S. & Das, S. S. Real-world evidence of glycemic control among patients with type 2 diabetes mellitus in India: the TIGHT study. *BMJ Open Diabetes Res. Care.* **7**(1), e000654 (2019). 31413840 10.1136/bmjdrc-2019-000654 PMC6673766

[7] American Diabetes Association Professional Practice Committee 6. Glycemic Goals and Hypoglycemia: Standards of Care in Diabetes—2024. Diabetes Care 1 January 2024; 47 (Supplement_1): S111–S125. 10.2337/dc24-S006 PMC10725808 38078586

[8] American Diabetes Association Professional Practice Committee 7 Diabetes Technology: Standards of Care in Diabetes—2024. Diabetes Care 1 2024; 47 (1): S126–S144. 10.2337/dc24-S007 PMC10725813 38078575

[9] Storz, M. A. & Ronco, A. L. Nutrient intake in low-carbohydrate diets in comparison to the 2020–2025 Dietary Guidelines for Americans: a cross-sectional study. *Br. J. Nutr.* **129**(6), 1–14 (2022). 35730148 10.1017/S0007114522001908 PMC9991840

[10] Janssen, et al. The impact and utility of very low-calorie diets: the role of exercise and protein in preserving skeletal muscle mass. *Curr. Opin. Clin. Nutr. Metab. Care* **26**(6), 521–527 (2023). 37724991 10.1097/MCO.0000000000000980 PMC10552824

[11] Vedel-Larsen, E. et al. Major rapid weight loss induces changes in cardiac repolarization. *J Electrocardiol* **49**, 467–472 (2016). 26925492 10.1016/j.jelectrocard.2016.02.005

[12] Berry, S. E. et al. Human postprandial responses to food and potential for precision nutrition. *Nat. Med.* **26**(6), 964–973 (2020). 32528151 10.1038/s41591-020-0934-0 PMC8265154

[13] Goldenberg, J. Z. et al. Efficacy and safety of low and very low carbohydrate diets for type 2 diabetes remission: Systematic review and meta-analysis of published and unpublished randomized trial data. *BMJ* **372**, m4743 (2021). 33441384 10.1136/bmj.m4743 PMC7804828

[14] Zeevi, D. et al. Personalized nutrition by prediction of glycemic responses. *Cell.* **163**, 1079–1094 (2015). 26590418 10.1016/j.cell.2015.11.001

[15] Mendes-Soares, H. et al. Model of personalized postprandial glycemic response to food developed for an Israeli cohort predicts responses in Midwestern American individuals. *Am J Clin Nutr.* **110**(1), 63–75 (2019). 31095300 10.1093/ajcn/nqz028 PMC6599737

[16] Seo, W., Lee, Y., Lee, S., Jin, S. & Park, S. A machine learning approach to predict postprandial hypoglycemia. *BMC Med. Inform. Decis. Mak.* **19**, 210 (2019). 31694629 10.1186/s12911-019-0943-4 PMC6833234

[17] Hadley F, Dunlap T, and Poon T. Precision treatment with machine learning and digital twin technology for optimal metabolic outcomes. US11185283B2. 2021.

[18] Fuller, A., Fan, Z., Day, C. & Barlow, C. Digital twin: Enabling technologies, challenges and open research. *IEEE Access* **8**, 108952–108971 (2020).

[19] Arambepola, C. et al. The impact of automated brief messages promoting lifestyle changes delivered via mobile devices to people with type 2 diabetes: A systematic literature review and meta-analysis of controlled trials. *J. Med. Internet Res.* **18**, e86 (2016). 27095386 10.2196/jmir.5425 PMC4873307

[20] Wallace, T. M., Levy, J. C. & Matthews, D. R. Use and abuse of HOMA modeling. *Diabetes Care* **27**, 1487–1495 (2004). 15161807 10.2337/diacare.27.6.1487

[21] Draznin, B. et al. Glycemic targets: Standards of medical care in diabetes-2022. *Diabetes Care* **45**, S83–S96 (2022). 34964868 10.2337/dc22-S006

[22] Battelino, T. et al. Clinical targets for continuous glucose monitoring data interpretation: Recommendations from the international consensus on time in range. *Diabetes Care* **42**, 1593–1603 (2019). 31177185 10.2337/dci19-0028 PMC6973648

[23] Hallberg, S. J. et al. Effectiveness and safety of a novel care model for the management of type 2 diabetes at 1 year: An open-label, non-randomized, controlled study. *Diabetes Therapy.* **9**, 583–612 (2018). 29417495 10.1007/s13300-018-0373-9 PMC6104272

[24] Look AHEAD Research Group. Reduction in weight and cardiovascular disease risk factors in individuals with type 2 diabetes: one-year results of the look AHEAD trial. *Diabetes care.* **30**(6), 1374–1383 (2007). 17363746 10.2337/dc07-0048 PMC2665929

[25] Lean, M. E. et al. Primary care-led weight management for remission of type 2 diabetes (DiRECT): An open-label, cluster-randomised trial. *Lancet* **391**, 541–551 (2018). 29221645 10.1016/S0140-6736(17)33102-1

[26] Saslow, L. R., Summers, C., Aikens, J. E. & Unwin, D. J. Outcomes of a digitally delivered low-carbohydrate type 2 diabetes self-management program: 1-year results of a single-arm longitudinal study. *JMIR Diabetes.* **3**(3), e12 (2018). 30291081 10.2196/diabetes.9333 PMC6238840

[27] Apolzan J.W. et al. A Scalable, Virtual Weight Management Program Tailored for Adults with Type 2 Diabetes: Effects on Glycemic Control. *Nutrition & Diabetes* 2023. 10.1038/s41387-023-00234-6 PMC10079927 37024467

[28] Mottalib, A., Sakr, M., Shehabeldin, M. & Hamdy, O. Diabetes remission after nonsurgical intensive lifestyle intervention in obese patients with type 2 diabetes. *J. Diabetes Res.* **2015**, 468704 (2015). 26114120 10.1155/2015/468704 PMC4465710

[29] Saboo, B. et al. Time-in-range as a target in type 2 diabetes: An urgent need. *Heliyon.* **7**(1), e05967 (2021). 33506132 10.1016/j.heliyon.2021.e05967 PMC7814148

[30] Deshmane, A. R. & Muley, A. Quality of life and its association with time in range among people with type 2 diabetes mellitus following different dietary interventions: A crossover clinical trial. *Cureus.* **16**(4), e57624 (2024). 38707009 10.7759/cureus.57624 PMC11069457

[31] Luis Zagury, R. et al. Randomized clinical trial to evaluate the effect on postprandial glycemia of Nutren Control®, a glycemia-targeted specialized supplement, compared to standardized breakfast in patients with type-2 diabetes: the CONTROL DIABETES study. *Nutrición Hospitalaria*10.20960/nh.04204 (2023). 10.20960/nh.04204 36602126

[32] Oliveira, B. F. et al. Impact of a low-carbohydrate compared with low-fat breakfast on blood glucose control in type 2 diabetes: a randomized trial. *Am. J. Clin. Nutr.* **118**(1), 209–217 (2023). 37257563 10.1016/j.ajcnut.2023.04.032

[33] Osugi, K. et al. Association between low-carbohydrate diets and continuous glucose monitoring-derived time in ranges. *J Diabetes Investig.* **14**(5), 659–668 (2023). 38078864 10.1111/jdi.13999 PMC10119912

[34] Mahmoudi, Z., Del Favero, S., Jacob, P. & Choudhary, P. Toward an optimal definition of hypoglycemia with continuous glucose monitoring. *Comput. Methods Progr. Biomed.* **209**, 106303 (2021). 10.1016/j.cmpb.2021.106303 34380077

